# Laser-Inscribed Stress-Induced Birefringence of Sapphire

**DOI:** 10.3390/nano9101414

**Published:** 2019-10-03

**Authors:** Hua Fan, Meguya Ryu, Reo Honda, Junko Morikawa, Zhen-Ze Li, Lei Wang, Jovan Maksimovic, Saulius Juodkazis, Qi-Dai Chen, Hong-Bo Sun

**Affiliations:** 1 State Key Laboratory of Integrated Optoeletronics, College of Electronic Science and Engineering, Jilin University, Changchun 130012, China; fanhua17@mails.jlu.edu.cn (H.F.); zhenze_lee@163.com (Z.-Z.L.); leiwang1987@jlu.edu.cn (L.W.); 2 School of Materials and Chemical Technology, Tokyo Institute of Technology, Meguro-ku, Tokyo 152-8550, Japan; ryu.m.ab@m.titech.ac.jp (M.R.); leo_0520@icloud.com (R.H.); morikawa.j.aa@m.titech.ac.jp (J.M.); 3 Tokyo Tech World Research Hub Initiative (WRHI), School of Materials and Chemical Technology, Tokyo Institute of Technology, 2-12-1, Ookayama, Meguro-ku, Tokyo 152-8550, Japan; 4 Centre for Micro-Photonics, Faculty of Science, Engineering and Technology, Swinburne University of Technology, Hawthorn, VIC 3122, Australia; jmaksimovic@swin.edu.au; 5 Melbourne Centre for Nanofabrication, ANFF, 151 Wellington Road, Clayton, VIC 3168, Australia; 6 State Key Laboratory of Precision Measurement Technology and Instruments, Department of Precision Instrument, Tsinghua University, Beijing 100084, China; hbsun@jlu.edu.cn

**Keywords:** femtosecond laser, birefringence, stress, sapphire

## Abstract

Birefringence of $3\times 10^{-3}$ is demonstrated inside cross-sectional regions of 100 μm, inscribed by axially stretched Bessel-beam-like fs-laser pulses along the c-axis inside sapphire. A high birefringence and retardance of λ/4 at mid-visible spectral range (green) can be achieved using stretched beams with axial extension of 30–40 μm. Chosen conditions of laser-writing ensure that there are no formations of self-organized nano-gratings. This method can be adopted for creation of polarization optical elements and fabrication of spatially varying birefringent patterns for optical vortex generation.

## 1. Introduction

Three-dimensional (3D) structuring of materials with a high refractive index at sub-wavelength resolution has promise to advance the field of photonic crystals (PhC) and the integration of PhC into photonic chips [1,2,3,4,5,6,7,8,9,10,11]. Femtosecond laser micro/nano-fabrication as the no contact method can directly pattern sub-wavelength ripples [12,13]). A 3D nonlinear PhC has been successfully fabricated inside lithium niobate using femtosecond laser [14]. However, this is still a challenging task [15] to deliver a close to diffraction-limited focusing at arbitrary depths required for the 3D patterning when using Gaussian-like laser pulses [16]. Compensation of spherical aberrations can be successfully achieved for laser-writing in high refractive index materials and large depths [17]. In this study, we enhance (instead of compensating [17]) the spherical aberration by tailoring axial light intensity to be stretched along the propagation direction and to form a Bessel-beam-like axial intensity profile. Similar techniques are used for 3D patterning inside high refractive index materials [5,6,8,18]. We use stretched pulses to control 3D structuring and dielectric permittivity change over tens-of-micrometers along the entire typical length of fs-laser pulses of 100–300 fs.

Permittivity $\varepsilon =n^{2}$ changes between the laser-inscribed region $\varepsilon_{1}$ (refractive index $n_{1}$) with width $t_{1}$ separated with a host material of permittivity $\varepsilon_{2}$ and width $t_{2}$, creates an artificial uniaxial form-birefringent structure with $\varepsilon_{e}-\varepsilon_{o}>0$, where e,o denotes extraordinary and ordinary beams polarized ‖ and ⊥ to the optical axis *OA*, respectively. Controlling the width of $t_{1}$, its period $t_{1}+t_{2}$ and depth *d* (along the light propagation) are essential parameters to an engineer’s optical elements for polarization control. Stress-induced birefringence is a well-used phenomenon to create artificial birefringent materials and used as a method for material inspection and characterization [19,20]. Generally, the inverse permittivity tensor is $\Delta \left(1/\varepsilon_{ij}\right)=P_{ijkl}\partial_{k}u_{l}$, where $P_{ijkl}$ is the fourth-rank photoelasticity tensor, $\partial_{k}u_{l}$ is the gradient of the displacement from equilibrium with $u_{l}$ being the linear displacement from equilibrium and $\partial_{k}$ denotes differentiation with respect to the Cartesian coordinates. Birefringence induced by stress and expanding beyond laser-structured regions is promising for polarization micro-optics, especially when the laser modified regions are not used for optical functions due to considerable light scattering losses.

In any type of material, the modification of adjacent regions with resolution smaller than the wavelength can be used to change the effective refractive index which for extraordinary $n_{e}$ (along the optical axis) and ordinary $n_{o}$ indices depend on the volume fraction f=t/Λ [21]:

(1) $$n_{e}=\sqrt{\frac{n_{1}^{2}n_{2}^{2}}{fn_{2}^{2}+\left(1-f\right)n_{1}^{2}}};\;\;\;n_{o}=\sqrt{fn_{1}^{2}+\left(1-f\right)n_{2}^{2}},$$

where $n_{1}$ and $n_{2}$ are the indices of the host and laser-inscribed regions, respectively. If modification of a silica host $n_{1}=1.40$ becomes $n_{1}=1.45$, the form birefringence would only reach maximum of $\Delta n=n_{e}-n_{o}=-8.8\times 10^{-4}$ when f=0.5, i.e., t=Λ/2. Hence, for a considerable phase delay required for λ/4 or λ/2 polarization optics, a tens-of-μm-long axial modification *d* would be required for engineering the retardance Δn×d. Moreover, a well-controlled laser inscription method developed here is required to reach the optimal conditions of f=0.5 for the shortest modification *d* at the chosen wavelength of operation (the strongest birefringence Δn). Such flexibility is currently not available for fabrication of micro-optical elements.

Here, we show that single nanolines array with controllable separation inscribed in crystalline sapphire (along *c*-axis) can reach retardance of λ/4 (π/2 in phase) for visible wavelengths using a simple approach to generate femtosecond pulses with an axially extended Bessel-like intensity distribution using a spatial light modulator (SLM).

## 2. Experimental

C-cut sapphire samples of 0.5 mm thickness were used for laser inscription. Sapphire has one of the highest Young moduluses of Y=400 GPa and can withstand high pressures strongly localized inside the crystal, as our earlier studies as shown in our earlier studies [22]. The stress-induced birefringence inside the laser-structured region patterned with single pulse irradiation reached $\Delta n\approx 1\times 10^{-3}$ and the pressure was estimated to reach 1.3 GPa. At ∼2 GPa, micro-cracks were developed when Gaussian fs-laser pulses were used in c-cut sapphire [22].

In this study, a fs-laser beam at second harmonic λ=515 nm wavelength and pulse duration of $t_{p}=280$ fs (Pharos, Light Conversion) was reflected from a phase mask designed on a spatial light modulator (SLM) and directed onto a tight focusing objective lens for laser structuring. Typical pulse energy used for stress-induced birefringent gratings was $E_{p}=574$ nJ (at focus) to inscribe single modification lines inside sapphire at laser repetition rate of 10 kHz at a beam scanning speed of 0.1 mm/s (if not specified otherwise). The focal diameter was ∼465 nm which can be calculated approximately using the formula d=1.22λ/NA. The phase mask pattern on the SLM was selected to create close-to-linear intensity distribution along the propagation direction and was characterized by a stretch factor $f_{st}$. For the $f_{st}=0$ the spherical aberration was compensated at the position of the Gaussian beam, while the largest value of $f_{st}=12$ was at the maximum stretch to obtain a linear intensity distribution over the entire pulse length $ct_{p}/n$, here $t_{p}$ is the pulse duration. The inscribed structure was approximately 10–15 μm below the sample surface. A laser-structured sapphire sample was cleaved to expose the structured regions and was etched in 20% HF for 60 min before SEM observation.

## 3. Results and Discussion

By rational choice of repetition rate and scanning speed, it was possible to create a single line without movement of the sample (or beam). By scanning the beams linearly modified regions, lengths of tens-of-micrometers were recorded. For the most axially stretched intensity distribution, single lines were recorded without usual formation of nanogratings [4,23]. First, we present structural characterisation of inscribed modified lines at a wider range of parameters and subsequently present results of optical characterisation of patterns which can deliver a λ/4 waveplate performance.

### 3.1. Direct Write of Nano-Planes

**Figure 1:**
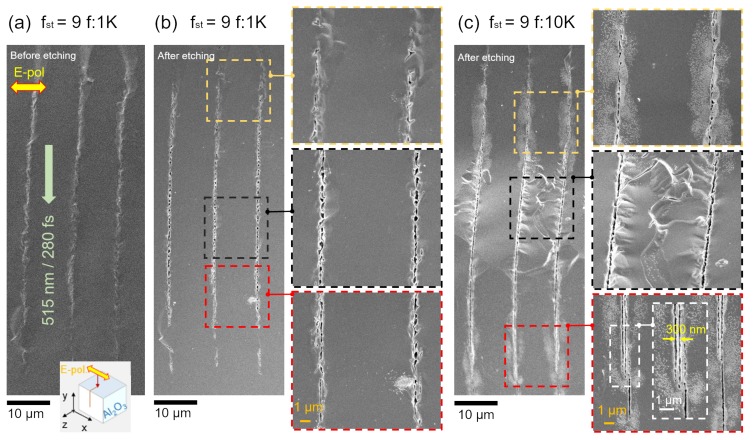
Inscription of sapphire with Bessel-beam at different stretch factors $f_{st}$ (0 corresponds to the spherical aberration compensation while the largest value of 12 was for the maximum stretch to obtain a linear intensity distribution over the entire pulse length $ct_{p}/n$, here $t_{p}$ is the pulse duration). SEM side-view images of as fabricated sample after breaking it on a xy-plane (see the inset) (**a**) and after wet etching in HF 20% vol. for 60 min (**b**,**c**) with same single pulse $E_{p}=847$ nJ at different pulse frequency: 1 kHz (**a**,**b**), 10 kHz (**c**); the beam was scanned at $v_{s}=0.5$ mm/s along *z*-axis. Some lines do not appear straight in SEM images due to the uneven surface of the cleaved region.

Inscription of long axial modifications in a crystalline sapphire were made by focusing onto a c-plane sample. Typical results of laser inscription are summarized in Figure 1. For same stretch factors $f_{st}=9$, different pulse frequency was used to study the evolutionary process of the femtosecond laser irradiated area (Figure 1b,c). The property of the irradiated (modified) area has been demonstrated in our previous work, which present the phase transition of the modified area from crystalline to amorphous in sapphire after irradiated by femtosecond laser [12]. 20% HF solution was employed due to the very high contrast of etching between crystalline sapphire and laser amorphized regions (∼$10^{4}$ etching selectivity) such as those seen previously when using silica and boro-silicate glasses [24,25,26,27,28]. Etching up to 0.8 mm into the depth of sapphire sample (along *z*-axis; inset in Figure 1a) was observed when continuous inscriptions of strongly modified regions were formed at large frequency.

For smaller pulse number and the same $f_{st}$, the individual laser damaged nano-regions, which were not initially interconnected into a single line, were observed connected after wet etching. With an increasing number of pulses (larger repetition rate or slow scanning), those single damage regions formed a line, which was further revealed after etching.

The longest inscription of modifications of up to 60 μm along the propagation direction (on *y*-axis) were inscribed with ∼850 nJ/pulse energy at $f_{st}=9$, f=10 kHz repetition rate and scanning speed of $v_{s}=0.5$ mm/s along *z*-axis (Figure 1a). At these conditions approximately $n=d/v_{s}/f\approx 46$ pulses were accumulated over the diameter of the focal spot (∼465 nm). Due to the large pulse energy, gratings with period of ∼300 nm were formed in the modified region as revealed after wet etching (inset in Figure 1c). The incident and scattered/reflected light from the strongly excited region interfere and form nanograting patterns as was described in ref. [29]. Near-sub-wavelength structures with a period of λ/n=515nm/1.45=355 nm were revealed in the strongly absorbing regions where the pulse-induced permittivity became metal-like.

**Figure 2:**
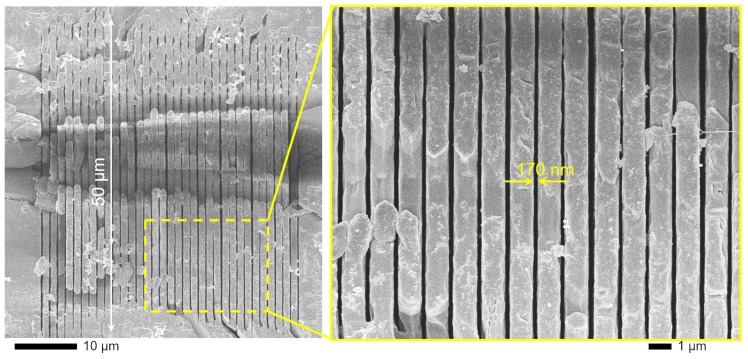
SEM images of the highest resolution single-line pattern inscribed in sapphire at the largest stretch factor $f_{st}=12$; conditions as in Figure 1a. The sample was cleaved (image plane) at some depth along the inscribed pattern and wet-etched for the SEM observation. The single line was inscribed by Bessel-like beam scanning without formation of periodicity nano-gratings ($f_{st}\leq 9$) nor transitional irregular pattern at low frequency and high pulse energy (Figure 1b). Wet-etched patterns reached aspect ratio 50/0.2=250.

As shown in Figure 2, an array of uniform non-period nanolines with 1μm interspace was inscribed by stretched fs-laser pulse (energy 574 nJ, scanning speed 0.1 mm/s). The width and length of the single nanoline after etching in HF solution were 50μm and ∼170 nm, respectively. The aspect ratio of the lines can reach 250, which pave the way to inscribe a strong and tunable birefringence and retardance in sapphire.

The use of an even distribution of pulse energy along the propagation axis can be applied in generation of high pressure and temperature phases of materials due to better energy delivery via resonant absorption [30]. The proposed phase control using SLM can, in principle, be adopted for experiments exploring a temporal evolution of fs-laser pulse-induced micro-explosions using femtosecond X-ray pulses of a free electron laser (FEL) for probing, while coaxially propagating Bessel-like beams [31] can be used for optically triggered micro-explosions. The current study confirms formation of an amorphous phase of sapphire which is typical in conditions of high pressure [32].

### 3.2. Engineering of Birefringence

**Figure 3:**
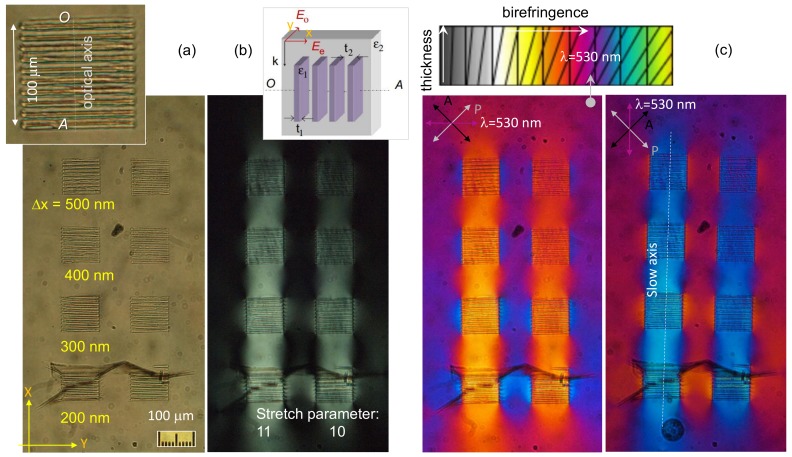
Optical (**a**), cross-polarized (**b**) and color-shifted birefringence with λ=530 nm waveplate at two orientations (**c**) images of the sample between polarizer (P) and crossed-oriented analyzer (A). The inset in (**a**) shows the pattern of 10 μm inscribed regions with nano-planes with separation of Δx. The form-birefringent pattern of negative uniaxial crystal $\Delta x=t_{1}+t_{2}$ and the orientation of ordinary and extraordinary fields $E_{o,e}$; *OA* is the optical axis (inset in (**b**)). The stretch parameter of 11 corresponds to ∼40μm axial extent of the laser-inscribed region, 10 corresponds to ∼30μm. The Michel-Levy birefrincence color chart is shown in the inset of (**c**).

Optical characterization of laser-inscribed gratings are shown in Figure 3. Gratings with Λ=10μm period were inscribed with duty cycle of 0.5, i.e., 10 μm were inscribed with a separation of Δx ranging from 170 nm to 500 nm between two single nanolines. The footprint of the gratings were 100×100μm${}^{2}$, an acceptable size for many applications in the field of micro-optical elements.

Single lines without the formation of self-organized nano-gratings were inscribed by stretching the incoming fs-laser pulses. The stretch factors of 10 and 11 corresponded to a single line (a plane under scanning) inscription for 30 μm and 40 μm, respectively. Inspection of the laser-inscribed regions with scanning electron microscopy (SEM) revealed the width of the structurally modified lines corresponding to ∼170 nm (Figure 2). When those modifications were written with Δx=200 nm separation, cracks formed during the laser-writing (Figure 3); however, for larger separations the gratings were stable. The writing depth was approximately 10μm below the surface and extending into the sample. Strong stress-induced birefringence was observed inside the gratings in the regions without laser damage as well as between the gratings as revealed by cross-polarized imaging.

To determine the sign of refractive index change $\Delta n=n_{e}-n_{o}$, a λ-waveplate at 530 nm was inserted at 45${}^{\circ }$ in respect to the orientation of polarizer and analyzer. In this setting, Michael-Levy color charts can be used to determine color changes corresponding to +|Δn| and −|Δn| (Figure 3c). For the λ-plate of 530 nm wavelength oriented vertically, the blue color indicates the stress-induced regions. Since the blue color on the Michael-Levy chart corresponds to the higher absolute birefringence and the orange to the lower, the change Δn has to be negative $n_{e}>n_{o}$ where $n_{o}$ is refractive index of the ordinary beam (perpendicular to the optical axis *OA*). The slow axis of the form-birefringent pattern (grating) is along the vertical direction (Figure 3c) and the refractive index is $\Delta n=n_{\|}-n_{⊥}=n_{e}-n_{o}>0$. Hence, the form-birefringent structure acts as a negative uniaxial crystal.

**Figure 4:**
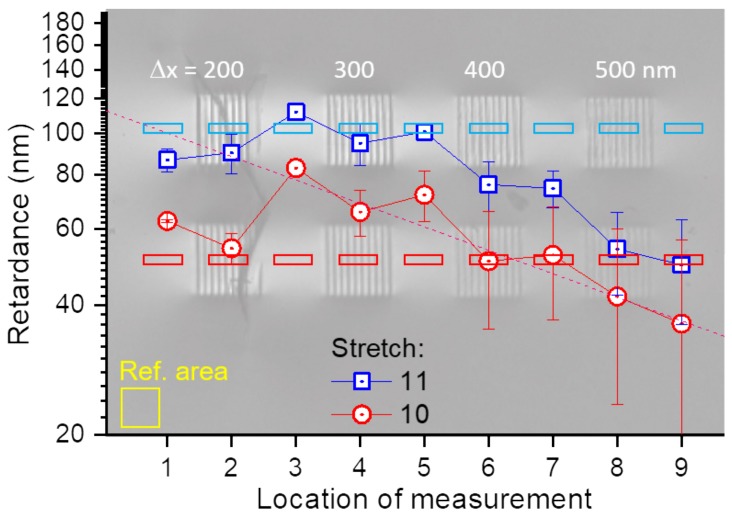
Retardance |Δn|d measured at several wavelengths from 475 nm to 650 nm with 10-nm-bandpass filters. Sample was fs-laser-inscribed at pulse energy $E_{p}=574$ nJ; sample is shown in Figure 3. Rectangular regions of interest (ROIs) show locations from where an average retardance was measured. Two lines of gratings with different stretch factors of 11 (the length of inscribed line d=40μm) and 10 (d=30μm) were analyzed using liquid crystal compensator [33]. Note logarithmic ordinate was used to reveal single exponential decay of retardance with Δx.

To determine birefringence Δn(λ) at several wavelengths, a recently developed method was used for measuring [33]. A single wavelength measurement of birefringence (as for example in the popular Abrio tool) leaves an ambiguity of the true Δn value due to a possible 2π folding of the phase and is avoided by carrying out measurements at several wavelengths in our method [33]. The measurement of Δn was made at five different wavelengths while using narrow 10-nm-bandpass filters spanning the visible spectral range of 475–650 nm. A linear fit through the origin point in the retardance (Δn×d [nm]) vs. wavenumber (1/λ) plot was obtained with a single slope which defines the Δn averaged over the tested spectral range [33]. Figure 4 shows the experimentally determined retardance for the grating patterns. Regions of interest (ROIs) were set to average retardance on the grating and in its vicinity. The error bars are the max and min retardance measured in the ROIs. The highest Δn×d was observed in between gratings and was scaling with separation Δx between nano-planes. For the largest retardance of 112 nm and stretch parameter of 11 (d=40μm), $\Delta n=2.8\times 10^{-3}$. Only slightly smaller birefringence was determined for the stretch factor of 10 and 30-μm-long inscribed gratings (Figure 4).

**Figure 5:**
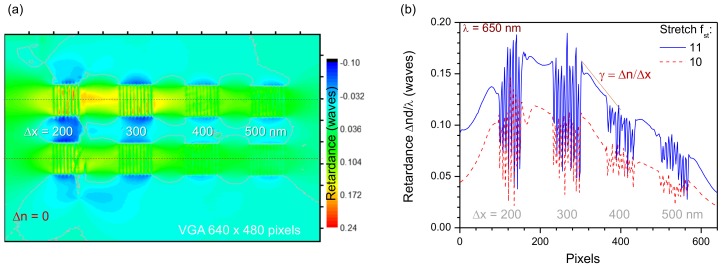
Retardance |Δn|d/λ [waves] measured at 650 nm; sample are shown in Figure 3. (**a**) Retardance map calculated at a single pixel level for VGA 640 × 480 pixel area. The δn=0 contour lines are shown to distinguish regions affected by stress-induced birefringence; the maximum was 0.22. Horizontal single-pixel cross sections are plotted in (**b**). The slope of retardance $\gamma =4.8\times 10^{-4}/$pixel or $(3.27\times 10^{-4})/\mu$m at the used magnification was achieved. One pixel corresponds to 1.4 μm in the image while the optical resolution for the NA=0.2 lens was 0.61λ/NA=2μm.

Even the larger Δn values were observed inside gratings in the 10-μm-wide openings reaching 22% retardance at the longest wavelength of 650 nm, selected for the measurement of birefringence (Figure 5a). For shorter wavelengths in visible range, a λ/4 waveplate condition was achieved by direct write of nano-inscribed modifications without changing the axial position of the modified region during inscription. Single-pixel cross-sections (Figure 5b) of the retardance maps show difference in the gradient of Δn between laser-inscribed regions. By placing a few regions of nano-planes at different depths from the surface, it should be possible to fabricate λ/2 waveplate retarders. Stress-induced regions between larger extended laser-structured patterns should allow reduction of light scattering observed from laser-inscribed areas which are used for fabrication of optical elements [4,34].

**Figure 6:**
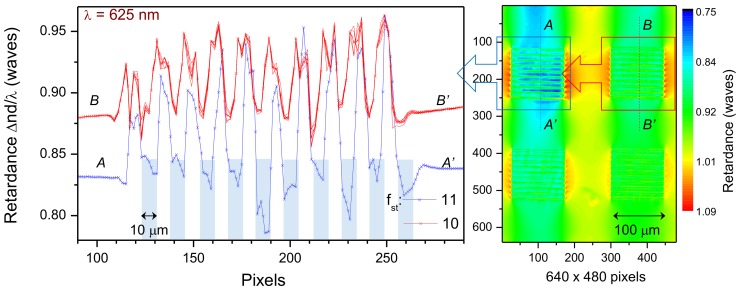
Retardance |Δn|d/λ [waves] measured at 625 nm with higher resolution NA=0.4; sample are shown in Figure 3 and Figure 5. Retardance map calculated at a single pixel level. Cross sections for two regions inscribed with stretch factors $f_{st}=11$ and 10 are shown as one-pixel line for Δx=200 nm; for $f_{st}=10$ five separate lines and their average (× marker in red line) are plotted. The optical resolution for the NA=0.4 lens was 0.61λ/NA=0.95μm. Rectangular markers show positions of the inscribed regions for the $f_{st}=11$ grating.

Optical resolution of large area birefringence mapping (Figure 5) have provided only a couple of points measured per 10 μm regions inside the grating. It illustrates a 0.1λ modulation of retardance. Measurements at approximately twice a higher resolution (Figure 6) confirmed the modulation amplitude of retardance shown in Figure 5. Retardance at 625 nm wavelength (Figure 6) is shown in relative units of waves since the reference retardance required to make calibration was not possible to measure from the stress-free region on the same image (as area “Ref.” in Figure 3). The cross sections of the measured retardance map clearly shows that stress-induced phase delay between inscribed 20-μm period and 0.5 duty cycle grating were clearly resolved between the laser-inscribed regions with axial extension tens-of-μm.

## 4. Conclusions and Outlook

We demonstrate λ/4 phase retardance at visible wavelengths in sapphire recorded by direct write of nano-inscribed modifications tens-of-micrometers long. This modality of laser structuring opens a flexibility in stress-induced optical element fabrication and eliminates light scattering since the regions of tailored birefringence are outside of laser-structured regions. Patterns of tailored birefringence can be produced at different depths along the light propagation direction or even in different micro-plates for the final optical element. One particular field of application can be spin-orbital couplers where spatially variant birefringence can be inscribed with complex 3D topology similar to the polymerized 3D couplers [35].
